# Pseudogenes and the associated ceRNA network as potential prognostic biomarkers for colorectal cancer

**DOI:** 10.1038/s41598-022-22768-y

**Published:** 2022-10-22

**Authors:** Zhuoqi Li, Jing Zhou, Liankun Gu, Baozhen Zhang

**Affiliations:** Key Laboratory of Carcinogenesis and Translational Research (Ministry of Education/Beijing), Division of Etiology, Peking University Cancer Hospital and Institute, Beijing, China

**Keywords:** Cancer, Biomarkers, Molecular medicine, Oncology

## Abstract

Colorectal cancer (CRC) is one of the most common and malignant carcinomas. Many long noncoding RNAs (lncRNAs) have been reported to play important roles in the tumorigenesis of CRC by influencing the expression of some mRNAs via competing endogenous RNA (ceRNA) networks and interacting with miRNAs. Pseudogene is one kind of lncRNA and can act as RNA sponges for miRNAs and regulate gene expression via ceRNA networks. However, there are few studies about pseudogenes in CRC. In this study, 31 differentially expressed (DE) pseudogenes, 17 DE miRNAs and 152 DE mRNAs were identified by analyzing the expression profiles of colon adenocarcinoma obtained from The Cancer Genome Atlas. A ceRNA network was constructed based on these RNAs. Kaplan–Meier analysis showed that 7 pseudogenes, 4 miRNAs and 30 mRNAs were significantly associated with overall survival. Then multivariate Cox regression analysis of the ceRNA-related DE pseudogenes was performed and a 5-pseudogene signature with the greatest prognostic value for CRC was identified. Moreover, the results were validated by the Gene Expression Omnibus database, and quantitative real-time PCR in 113 pairs of CRC tissues and colon cancer cell lines. This study provides a pseudogene-associated ceRNA network, 7 prognostic pseudogene biomarkers, and a 5-pseudogene prognostic risk signature that may be useful for predicting the survival of CRC patients.

## Introduction

According to the GLOBOCAN 2018 assessment on cancer incidence and mortality published by the International Agency for Research on Cancer, colorectal cancer (CRC) was classified with the third (10.2%) and second (9.2%) highest incidence and mortality rates respectively among all cancer types^1,2^. Unfortunately, the prognosis prediction of CRC remains pessimistic. Molecular biomarkers for diagnosis and prediction have great clinical significance with the development of precision medicine. The molecular mechanisms for the development of CRC are clinically important for the prognosis and treatment response of patients. In addition to the traditional genetic and epigenetic alterations of protein-coding genes, noncoding RNAs (ncRNAs) were considered to play important roles in regulating various biological behaviors, such as cell proliferation, metastasis, apoptosis, differentiation, etc.^3^. Many studies have discovered that endogenous long noncoding RNAs (lncRNAs) can act as sponges and competitively bind with RNAs in gene expression regulatory networks, which can influence cell fate decisions in cancer development^4^. NcRNAs are rising as biomarkers of CRC for diagnosis, prognosis, and even prediction of therapeutic effect^5^.

Competing endogenous RNAs (ceRNAs) can regulate each other at the post-transcription level by competing for shared miRNAs. CeRNA networks link the function of protein-coding mRNAs with ncRNAs such as miRNA, lncRNA, pseudogenic RNA and circular RNA^6^. Recently, the lncRNA related ceRNA crosstalk was highlighted in the CRC initiation and progression^7,8^. Pseudogenes may derive from gene mutations, or unfaithful gene duplications, or retrotransposition of processed mRNAs back into the genome. Accordingly, pseudogenes can be categorized into three types: (1) unitary pseudogenes, (2) duplicated or unprocessed pseudogenes and (3) processed or retrotransposed pseudogenes^9,10^. An increasing number of studies have shown that pseudogenes are involved in the occurrence and development of cancer through ceRNA networks. For example, the pseudogene PTENP1 could be targeted by multiple PTEN-targeting miRNAs and then regulate the protein level of PTEN^11^. PTENP1 could bind with miR-21, miR-200c, or miR-20a and regulate the expression of PTEN gene and further affect the development of hepatocellular carcinoma, endometrioid endometrial carcinoma, or breast cancer^12–14^. There are also a few studies on pseudogene function in CRC tumorigenesis and development. For example, the pseudogene DUXAP8 could promote colon cancer cell proliferation, migration and invasion by targeting tumor suppressor miR-577 and promote the expression of oncogene RAB14^15^. The pseudogene FLT1P1 could promote VEGFR1 and VEGF-A expression by interacting with miR-520a, thus contributing to CRC cell growth^16^.

In this study, we first comprehensively analyzed aberrantly expressed pseudogenes, miRNAs and mRNAs in the colon adenocarcinoma (COAD) dataset from The Cancer Genome Atlas (TCGA) and constructed a pseudogene-associated ceRNA network for CRC. We also discovered some novel pseudogenes and mRNAs that were significantly related to the overall survival of patients with CRC, and identified a five-pseudogene prognostic risk signature. More importantly, these results were validated in Gene Expression Omnibus (GEO) datasets and qRT-PCR experiments in our CRC samples.

## Results

### Identification of differentially expressed (DE) pseudogenes, miRNAs and mRNAs in CRC

**Figure 1:**
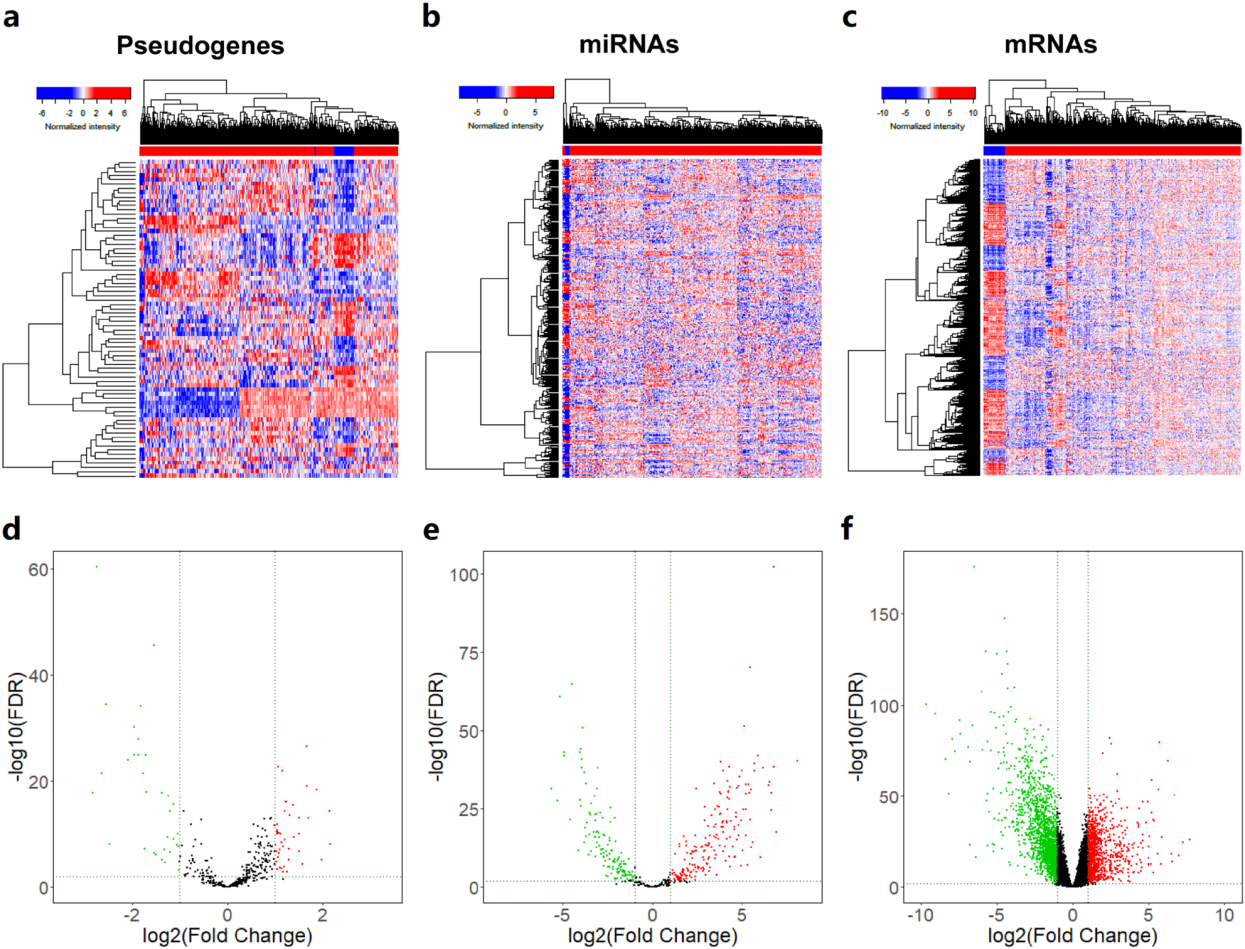
Differentially expressed RNAs from TCGA-COAD compared with adjacent normal tissues. (**a**–**c**) DE pseudogenes, miRNAs and mRNAs are hierarchically clustered by R software. The upper horizontal axis denotes the cluster analysis of each sample, blue indicates adjacent normal tissue and red indicates tumor samples. The left longitudinal axis indicated the cluster analysis of DE RNAs. The blue and red blocks represent relatively low and high expression respectively. (**d**–**f**) Each RNA analysis was plotted into the volcano map and the red dots represent the upregulated DE genes with log2FC ≥ 1 and adjusted *p* value FDR < 0.01, while the green dots represent downregulated genes with log2FC ≤ -1 and FDR < 0.01. FC, fold change. FDR, false discovery rate.

By using the edgeR package and the threshold set at FDR < 0.01 and |log2FC| ≥ 1, we made a comparison between the 469 primary CRC samples and 41 normal colon tissues and identified 74 DE pseudogenes (including 42 upregulated and 32 downregulated pseudogenes), 340 DE miRNAs (203 upregulated and 137 downregulated), and 2957 DE mRNAs (1128 upregulated and 1829 downregulated) in tumors. Heatmaps and volcano plots of the DE pseudogenes, miRNAs and mRNAs were generated by gdcHeatmap and gdcVolcanoPlot3 in the R platform, as shown in Fig. 1.

### Construction of the ceRNA network in CRC

**Figure 2:**
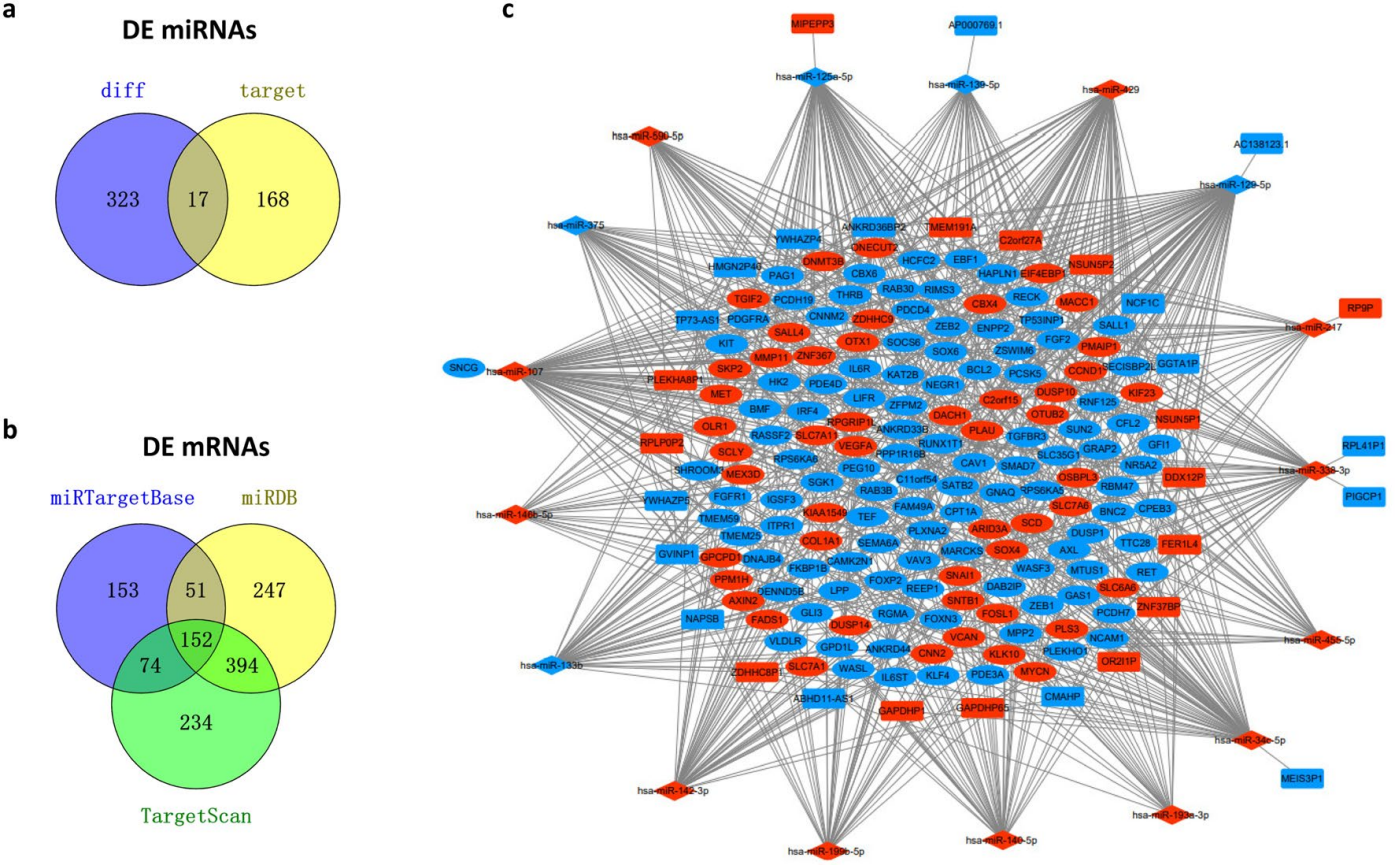
Construction of the ceRNA network for DE pseudogenes-miRNA-mRNA. (**a**, **b**) the overlapping DE miRNAs and mRNAs in different databases. (**c**) the ceRNA network. Round rectangles represent pseudogenes, diamonds represent miRNAs, and ellipses represent mRNAs. Blue represents downregulated genes, while red represents upregulated genes.

To better understand the interactions among these DE pseudogenes, DE miRNAs and DE mRNAs in CRC, we constructed a pseudogene-miRNA-mRNA related ceRNA regulatory network. First, we found that 31 of 74 DE pseudogenes could be targeted by 185 miRNAs by miRcode database. Of the 185 targeted miRNAs, only 17 miRNAs overlapped with the 340 DE miRNAs (Fig. 2a). Therefore the 17 miRNAs were selected to predict their target mRNAs through the miRTarBase, miRDB and TargetScan databases. There were 430, 844 and 854 mRNAs that could be targeted by the 17 miRNAs and overlapped with the DE mRNAs in the 3 databases. The 152 mRNAs presented in all three databases were selected to construct the ceRNA network (as shown in Fig. 2b). Finally, we incorporated the 31 pseudogenes, 17 miRNAs and 152 mRNAs to build the ceRNA network using Cytoscape software, and the visualized map was shown in Fig. 2c. The DE pseudogenes, miRNAs and mRNAs were listed in Supplemental Table 1.

### Identification of survival-related DE pseudogenes in the ceRNA network

**Figure 3:**
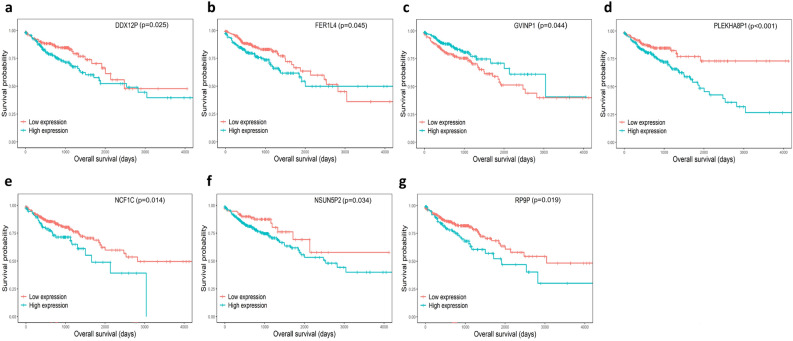
Kaplan–Meier curve analysis of DE pseudogenes in the ceRNA network. Seven pseudogenes were found to be significantly related to overall survival with *p* < 0.05. (**a**) DDX12P, (**b**) FER1L4, (**c**) GVINP1, (**d**) PLEKHA8P1, (**e**) NCF1C, (**f**) NSUN5P2, (**g**) RP9P.

To explore the prognostic value of the DE pseudogenes, miRNAs and mRNAs involved in the ceRNA network of CRC, we conducted Kaplan–Meier curve analysis using R software for CRC patients from the TCGA database. As shown in Fig. 3, 7 of the 31 pseudogenes had a significant relationship with overall survival (*p* < 0.05). Except that GVINP1 was positively associated with overall survival, the other six pseudogenes DDX12P, NCF1C, FER1L4, NSUN5P2, PLEKHA8P1 and RP9P were negatively associated with overall survival. Association analysis for the expression of these genes with clinicopathological factors of patients in TCGA-COAD showed that DDX12P, FER1L4, GVINP1, PLEKHA8P1 and RP9P were related to the T, N, M or pathologic stage (Supplemental Table 2).

**Figure 4:**
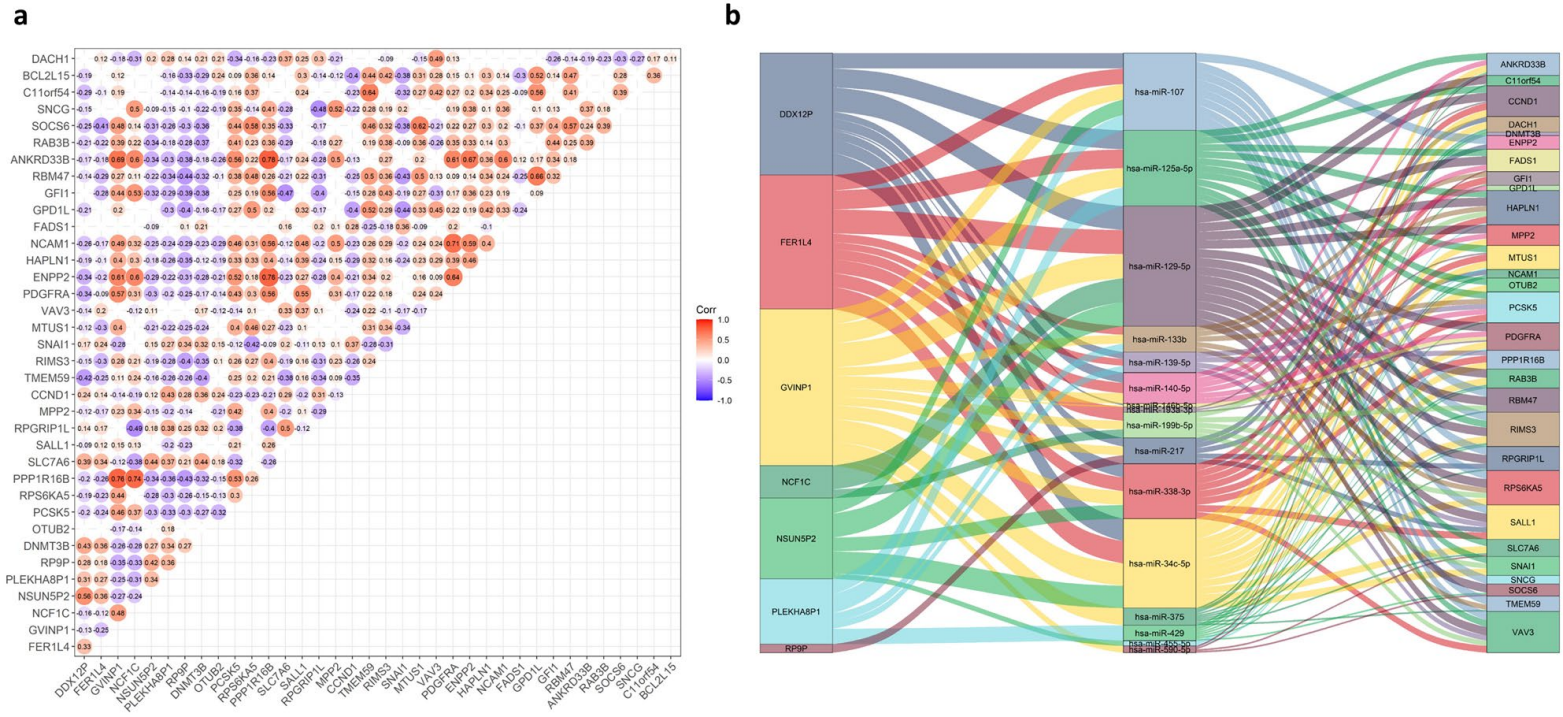
Survival-related 7 DE pseudogenes and ceRNA network. (**a**) Pearson correlation analysis of the 7 survival-related pseudogenes and 29 survival-related mRNAs with *p* < 0.05. Red presents a positive correlation and purple presents a negative correlation. (**b**) Sankey diagram showing interactions between the 7 pseudogenes and their matched miRNAs and mRNAs that were significantly related to survival. Each rectangle represents a gene, and the connection degree of each gene is visualized based on the size of the rectangle.

In addition, 4 of 17 DE miRNAs and 30 of 152 DE mRNAs in the above ceRNA network were significantly associated with the overall survival of patients with CRC (Supplemental Table 1 and Supplemental Figure 1 & 2). The Pearson correlation coefficient analysis between the survival-related pseudogenes and mRNAs was carried out by using the R package “ggcorrplot”. Figure 4a shows the 30 prognosis related DE mRNAs correlated with the 7 DE pseudogenes. For example, the pseudogene DDX12P and FER1L4 were significantly positively correlated with the DNMT3B gene, GVINP1 and NCF1C were highly correlated with the PPP1R16B, PDGFRA, ENPP2, ANKRD33B and SOCS6 genes, PLEKHA8P1 and RP9P were coexpressed with the CCND1 and SNAI1 genes. Furthermore, a Sankey diagram was constructed using the R package “ggalluvial” to visualize the interaction network among the 7 prognosis-related pseudogenes and the 30 prognosis-related mRNAs through binding with 16 DE miRNAs (Fig. 4b).

### Construction of the 5-pseudogene prognostic risk signature

**Figure 5:**
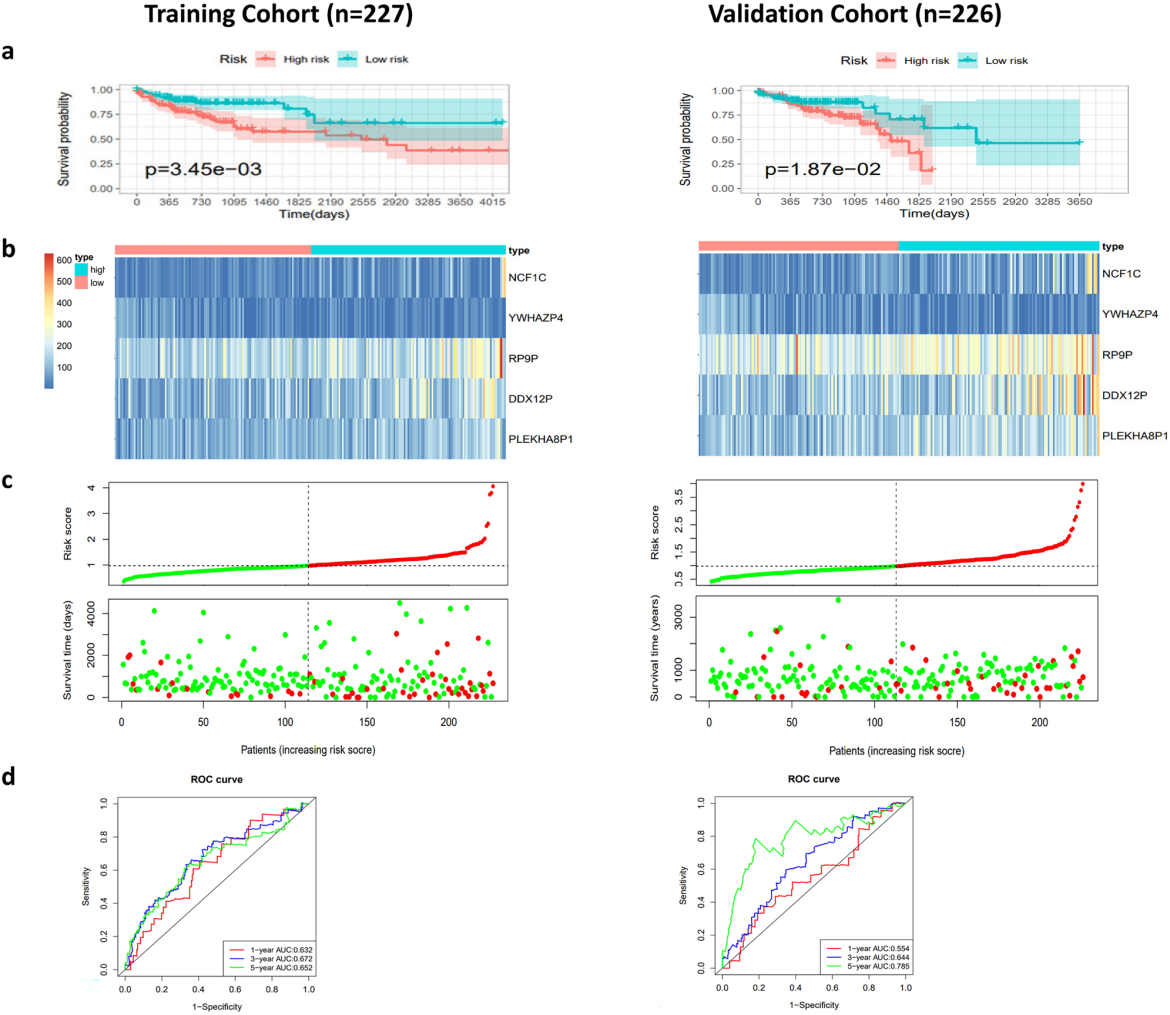
Characterization of the five-pseudogene risk signature in the ceRNA network in the training and validation cohorts. (**a**) Kaplan–Meier curves for high-risk and low-risk groups classified by the risk scores of this signature. (**b**) The expression profiles of the 5 pseudogenes of each sample. The value of risk increased gradually from left to right. (**c**) The risk score distributions and the survival status of CRC patients. The patients were ranked by risk score. (**d**) ROC curves for predicting the 1-, 3-, and 5-year survival of CRC patients according to risk scores.

To better understand the prognostic value of the aberrantly expressed pseudogenes in CRC, we calculated the risk scores of DE pseudogenes through multivariate Cox regression analysis based on TCGA samples. A total of 453 CRC patients were randomly divided into a training cohort (n = 227) and a validation cohort (n = 226), and no significant differences in the pathological characteristics were found between the two groups (Supplemental table 3). A remarkable prognostic risk model was constructed by multivariate Cox regression in the training cohort including four survival-related pseudogenes (NCF1C, RP9P, DDX12P and PLEKHA8P1) and one unrelated pseudogene (YWHAZP4) (Supplemental table 4). The risk scores were calculated using the formula as follows: risk score = (0.002045 × expression level of DDX12P) + (0.003879 × expression level of NCF1C) + (0.003856 × expression level of PLEKHA8P1) + (0.001913 × expression level of RP9P)-(0006358 × expression level of YWHAZP4). The training CRC patients were ranked by the risk score and divided into low-risk (n = 114) and high-risk (n = 113) groups. The Kaplan–Meier curve showed significantly poorer prognosis in the high-risk group than in the low-risk group (*p* = 0.0059) (Fig. 5a). According to the risk score heatmap, the expression levels of NCF1C, RP9P, DDX12P and PLEKHA8P1 were upregulated while the expression level of YWHAZP4 was decreased with increasing risk scores (Fig. 5b). The risk score distributions of CRC patients were shown in the high- and low-risk groups (Fig. 5c). The AUC curve was used to evaluate the efficacy to predict the 1-, 3-, and 5-year survival of CRC patients, and they were 0.632, 0.672 and 0.652 in the training cohort and 0.554, 0.644 and 0.785 in the validation cohort respectively (Fig. 5d). The results of the validation cohort were consistent with the results of the training cohort, which suggested the efficiency of this 5-pseudogene prognostic risk signature. In addition, after adjustment for age, gender and pathologic stage, the risk score was still an adequate prognostic indicator in multivariate analysis in both the training and validation cohorts (Supplemental Figure 3).

### Validation of the DE pseudogenes through a GEO dataset and our CRC samples

**Figure 6:**
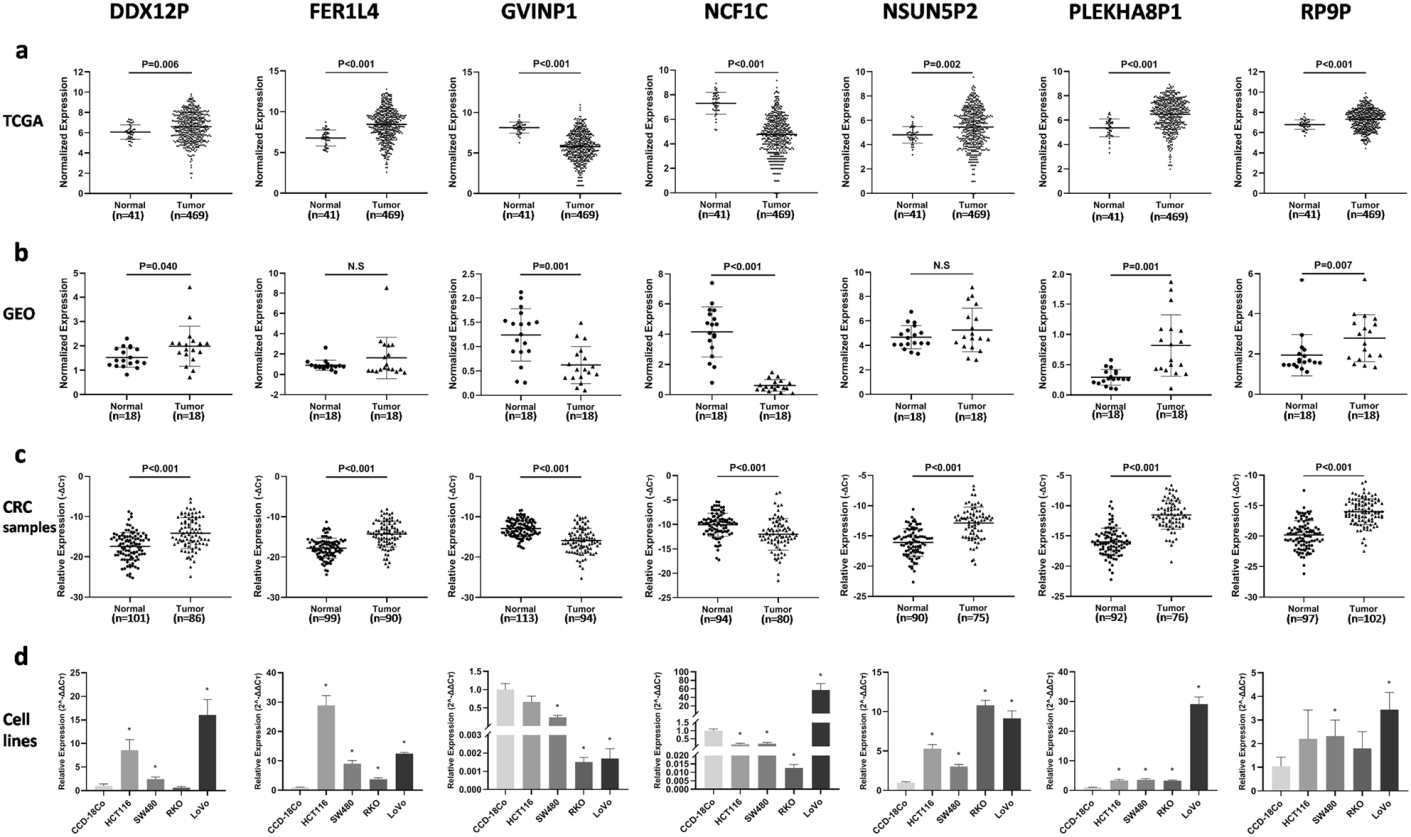
The expression levels of the 7 survival‐related DE pseudogenes in different datasets. (**a**) In normal colon tissues and colon cancer in TCGA. (**b**) Normal colon tissues and paired colorectal cancer in GSE50760 dataset of GEO. (**c**) In colorectal cancer tissues and paired normal tissues collected in our hospital, measured by qRT-PCR. (**d**) In one human normal colon fibroblastic cell line (CCD-18Co) and four human colorectal cancer cell lines (HCT116, SW480, RKO, LoVo), measured by qRT-qPCR. *p < 0.05. N.S, not significant.

To validate the expression alteration of the seven DE pseudogenes between normal and primary CRC tissues, we analyzed the 7 DE pseudogenes in a GEO dataset, CRC samples from our hospital and colon cell lines at the same time. The expression of all seven pseudogenes in 113 pairs of CRC samples from Peking University Cancer Hospital were measured by qRT-PCR. Except for GVINP1 and NCF1C, the expression of the other 5 prognosis-related DE pseudogenes (DDX12P, FER1L4, NSUN5P2, PLEKHA8P1 and RP9P) were higher in tumor tissues than in cutting edge normal tissues, which was totally consistent with the results of TCGA samples. Unfortunately, the expression levels of these 7 pseudogenes were not found to be related to overall survival in the 113 CRC samples. Five of the seven DE pseudogenes were also validated in 18 pairs of CRC samples from GSE50760. The expression of these pseudogenes was confirmed in the normal colon cell line CCD-18Co and 4 CRC cell lines (Fig. 6). In addition, the prognostic values of some individual pseudogenes were validated in TCGA-READ and GSE14333 datasets, results shown in Supplemental Figure 4.

### The function and pathway enrichment analysis of DE mRNAs

**Figure 7:**
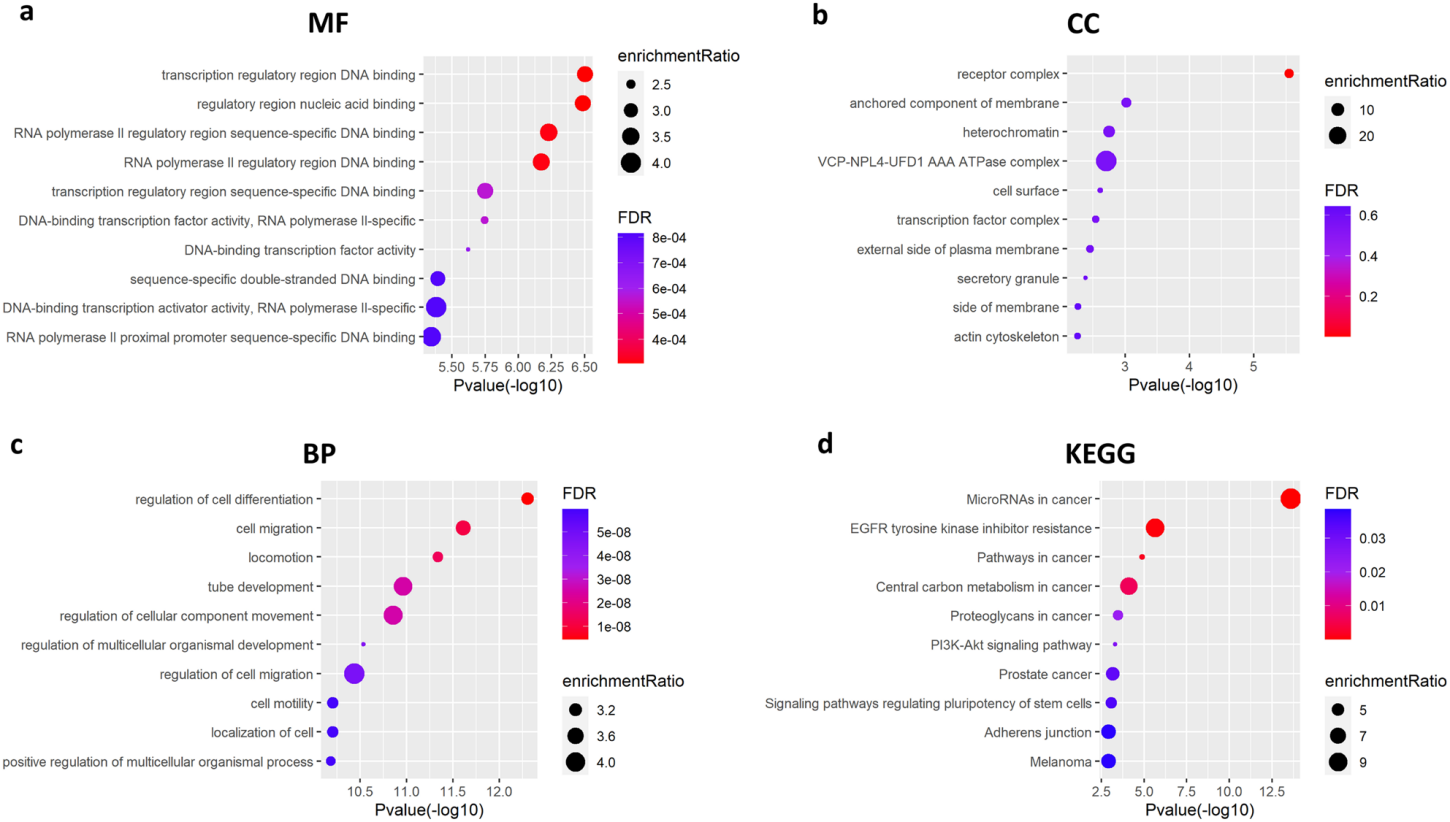
GO annotation and KEGG pathway enrichment analysis of the DE mRNAs in the ceRNA network. The top 10 enriched GO (**a**) MF, (**b**) CC and (**c**) BP terms as well (**d**) KEGG pathways. GO, gene ontology; KEGG, Kyoto Encyclopedia of Genes and Genomes; MF, molecular function; CC, cellular component; BP, biological process.

To explore the possible regulatory mechanisms of the prognosis-related DE pseudogenes in CRC, the 152 DE mRNAs involved in the pseudogene-miRNA-mRNA related ceRNA network were further selected for GO annotation and KEGG pathway enrichment analysis to analyze the possible functions and molecular pathways of these genes. The GO biological process (BP) analysis showed that the DE mRNAs were mainly involved in the regulation of cell differentiation, cell migration and locomotion (Fig. 7a). The cellular component (CC) analysis showed that many of these mRNAs might be components of the receptor complex (Fig. 7b). The molecular function (MF) analysis suggested that these mRNAs played roles in regulating the transcription of some genes because they were significantly associated with transcription regulatory region DNA binding, RNA polymerase II regulatory region DNA binding, regulatory region nucleic acid binding, DNA− binding transcription activator activity and so on (Fig. 7c). Moreover, KEGG pathway analysis revealed that these mRNAs had a clear relationship with cancer, as they were enriched in pathways such as microRNAs in cancer, EGFR tyrosine kinase inhibitor resistance, and pathways in several kinds of cancers (Fig. 7d).

## Discussion

Pseudogenes are abundant in the human genome and conventionally considered as nonfunctional “junk genes.” However, recent studies have revealed that pseudogenes are aberrantly expressed in cancers and may function in tumorigenesis through pseudogene-derived lncRNA transcripts. They may serve as ceRNAs by competitively binding to shared miRNAs, thus affecting both their cognate genes and unrelated genes and playing an important role in regulating ceRNA networks^17,18^. To deeply explore the underlying mechanisms of pseudogenes in CRC carcinogenesis and development and investigate novel candidate biomarkers for CRC diagnosis and prognosis prediction, in this study, we identified DE pseudogenes, miRNAs and mRNAs between CRC tumor tissues and adjacent normal tissues and constructed a ceRNA network. To the best of our knowledge, this report may be the first to describe the regulatory network among pseudogenes, miRNAs and mRNAs in CRC.

There are some evidences that the prognosis-related pseudogenes discovered in this study could regulate tumorigenesis and tumor development through ceRNA regulatory networks. For example, NSUN5P2 was found to be unfavorable for the prognosis of hepatocellular carcinoma through bioinformatic analysis^19^. PLEKHA8P1 was also implicated as an oncogene and prognosis-related gene in both colorectal and liver cancer^20,21^. These results are consistent with the results of our findings in CRC. Furthermore, FER1L4 was reported to be an oncogenic and adverse prognostic marker in pancancer, renal cancer and glioma^22–24^. Meanwhile, FER1L4 was also found to act as a tumor suppressor in prostate cancer, gastric cancer, hepatocellular carcinoma and colon cancer cells^25–28^. GVINP1 was downregulated in lung cancer and related to poor prognosis of patients with lung cancer^29,30^. However, for the remaining pseudogenes (DDX12P, NCF1C, RP9P, and YWHAZP4), there were no reports on their biological functions in cancers. In our study, we found that YWHAZP4 was a protective factor for CRC patients while DDX12P, NCF1C and RP9P were adverse factors for CRC patients. Thus, these five pseudogenes might be novel prognostic biomarkers for CRC.

The ceRNA network provided a way to uncover the underlying regulatory functions and mechanisms of pseudogenes in CRC. We found that the expression levels of survival-related pseudogenes were significantly correlated with many protein-coding genes that have been reported to be aberrantly expressed or mutated and play roles in tumorigenesis and progression in many kinds of cancers, such as DNMT3B, PDGFRA, SOCS6, SNCG, CCND1, and SNAI1^31–35^. The correlation analysis and Sankey diagram showed candidate miRNAs and the regulatory network among the pseudogenes, miRNAs and target genes. Some DE transcription factors (TFs) were involved in the ceRNA network might imply a complicated regulatory circuit. For example, SNAI1 and six of the seven prognostic pseudogenes could be targeted by 3 miRNAs (hsa-miR-125a-5p, hsa-miR-199b-5p, hsa-miR-34c-5p) in the ceRNA network. This indicates a potential clue to deeply explore the regulatory mechanism and biological functions of these pseudogenes. Notably, some of the features that different models of ceRNA predictions have highlighted the importance of the number of MREs on transcripts, the combinatorial effect of miRNA molecules, and the number of molecules. The significant cross-regulation may occur preferably when the stoichiometry of the interrelated ceRNA and miRNA falls in a narrow range of equivalence^36–38^. This important point requires special attention when the researchers are validating the crosstalk by experiment.

With the development of precision medicine, there is an increasing demand for finding prognostic biomarker. The transcriptional signatures of consensus molecular subtypes (CMSs) and colorectal cancer intrinsic subtypes (CRISs) have been proposed based on transcriptomics and have potential application for improving prognostic assignment^39,40^. Recent studies identified stromal genes’ expression might define poor-prognosis subtypes of colorectal cancer^41,42^. In this study, we selected and analyzed stromal and epithelial genes to explore the significance in the prognostic pseudogene model (as shown in Supplemental Figure 5). The expressions of stromal genes TGFB1 and SNAI1 were higher, the epithelial genes CDH1 and EPCAM were lower in the high-risk group than in the low-risk group. These results support the predictive potential of the constructed pseudogenes signature.

## Conclusion

In conclusion, this study provides a way to uncover the underlying regulatory functions and mechanisms of pseudogenes in CRC. Some novel potential diagnostic and prognostic biomarkers for CRC were discovered through identification of the 5-pseudogene signature and clinical analysis.

## Materials and methods

### TCGA data collection and processing

The RNA-seq data of 521 samples and miRNA-seq data of 465 samples with colon adenocarcinoma were retrieved from the TCGA data portal (https://portal.gdc.cancer.gov/). R software and the package GDCRNATools were applied to read the RNA-seq sample sheet and remove the repetitive samples and the samples that were not primary tumors. Finally, 469 primary CRC tumors and 41 normal tissues in total were collected. The RNA-seq data contained more than 60,000 genes including noncoding genes with the Ensembl Gene ID. For miRNAs, a matrix of 451 primary tumors and 8 normal tissues was built with the expression level of all the genes. The miRNA-seq data included more than 2,500 miRNAs with annotated miRNA IDs. In addition, the corresponding clinical information was downloaded. The sample sheets provided information on case ID, sample ID, sample type and clinical information such as race, ages, gender, pathologic stage, vital status, days to death or days to last follow up of the patients. This study was in accordance with the publication guidelines provided by TCGA (https://cancergenome.nih.gov/publications/publicationguidelines). All the packages and databases in the following analysis were well-established open data and require no further ethical approval.

### Identification of differentially expressed (DE) genes

The edgeR package was used to determine the DE pseudogenes, miRNAs and mRNAs between primary tumors and normal tissues, with the threshold setting at an adjusted *p* value < 0.01 and |log2-fold change (FC)| ≥ 1 based on all transcripts. Benjamini–Hochberg method was used to adjust the *p* value. Heatmaps and volcano maps of the DE pseudogenes, miRNAs and mRNAs were also generated using gdcHeatmap and gdcVolcanoPlot3 of the GDCRNATools package in the R platform.

### Construction of the ceRNA network

The miRcode database (http://www.mircode.org/) was used to predict the interactions between pseudogenes and miRNAs in the COAD dataset of TCGA. The miRNAs-targeted mRNAs were retrieved using the miRTarBase, miRDB and TargetScan databases, and only the miRNA-targeted mRNAs present in all three databases were included to construct the ceRNA network. Cytoscape 3.6.1 software (https://cytoscape.org/) was used to visualize the ceRNA network. The DE genes were the nodes and their interactions were the edges in the network. The Sankey diagram was constructed by using the “ggalluvial” and “ggplot2” packages in R software to show the interactions between the survival-associated pseudogenes and mRNAs, along with their matched miRNAs in the ceRNA network.

### Identification of a 5-pseudogene prognostic risk signature

The 453 CRC patients were randomly divided into a training cohort (n = 227) and a validation cohort (n = 226). Multivariate Cox regression analysis was performed to identify the prognostic model for the pseudogenes in the ceRNA network, and the risk score of the patients with CRC was calculated according to the expression level of the involved pseudogenes weighted by the regression coefficient (βpseudogenes), as follows: Risk score = expression of pseudogene1 × β1pseudogene1 + expression of pseudogene2 × β2pseudogene2 + ··· expression of pseudogeneN × βNpseudogeneN. The pseudogene prognostic model was constructed based on the training cohort and then confirmed in the validation cohort. According to the risk score of the prognostic model, the CRC patients were divided into two groups of low-risk and high-risk by the median risk score value. Then the Kaplan–Meier analysis was conducted by the R package “survival” to generate the overall survival (OS) curve for the two groups. ROC curve analysis was conducted by the package “survival ROC” to evaluate the accuracy of the prognostic model of 1, 3, and 5-year survival. In addition, a risk heatmap for the pseudogenes involved in the ceRNA network of the patients with CRC was plotted by the R package “pheatmap” combining the gene expression and clinical survival data.

### Survival analysis and correlation analysis

The R package “survival” was used for survival analysis for the DE RNAs involved in the ceRNA network and plotting Kaplan–Meier curves. The R package “ggcorrplot” was applied to perform Pearson correlation coefficient analysis between the survival-associated mRNAs and pseudogenes in the ceRNA network and carried out by the R function “cor_pmat”. The survival-associated pseudogenes were analyzed with clinical pathological characteristics of CRC patients using the chi-square test and t-test in SPSS 20.0 software. *p* < 0.05 was considered statistically significant.

### Validation with a GEO dataset

The GSE50760 and GSE14333 datasets were downloaded from the Gene Expression Omnibus (GEO, https://www.ncbi.nlm.nih.gov/geo/) database to validate the expression level of the survival-related genes in CRC and normal tissues. The GSE50760 dataset is an expression profiling by high throughput sequencing, containing RNA-seq data of 54 samples (normal colon, primary CRC, and liver metastasis) generated from 18 CRC patients^43^. The GSE14333 dataset contained the expression profiling by array of 290 primary colorectal cancers^44^. Student’s t-test was conducted for normally distributed data while the Mann–Whitney U-test was conducted for nonnormally distributed data in SPSS 20.0 software, with statistical significance assigned at *p* < 0.05.

### Quantitative real-time polymerase chain reaction validation

Quantitative real-time polymerase chain reaction (qPCR) was performed to detect the DE pseudogene expression in both colon cell lines and clinical samples. For cell lines, a human normal colon fibroblastic cell line (CCD-18Co) was purchased from ATCC and cultured in MEM plus 15% fetal bovine serum (FBS) and 100 U/mL penicillin/streptomycin (Gibco, USA), and four human colon cancer cell lines (HCT116, SW480, RKO and LoVo) were grown in RPMI 1640 medium with 10% FBS and 100 U/mL penicillin/streptomycin at 37 °C in a humidified incubator with 5% CO2. A total of 113 paired human CRC tumor and cutting edge tissues were collected and stored at − 80 °C in Peking University Cancer Hospital, China. Research protocols were approved by the Institutional Review Board of the Peking University Cancer Hospital and Institute. All patients in this study provided written informed consent.

Total RNA was extracted using the Direct-zol™ RNA MiniPrep kit (Zymo research, USA) according to the manufacturer's instructions. Then complementary DNA (cDNA) was synthesized using TransScript First-Strand cDNA Synthesis SuperMix (TransGen Biotech, China). Next, reverse transcription qPCR (RT-qPCR) was performed using the FastStart Universal SYBR Green Master Mix (ROX) (Roche, Germany) on an ABI-7500 Fast system (Applied Biosystems). GAPDH was used as the endogenous reference gene for the cultured cell lines, while ALU was used for tissues. The expression levels of the survival-associated pseudogenes in the ceRNA network were determined using the typical ΔΔCt method. The correlations with clinical pathological characteristics and survival were also analyzed with the chi-square test, Student’s t-test and Kaplan–Meier test in SPSS 20.0. *p* < 0.05 was considered statistically significant.

The following primer sequences were used in this study: DDX12P, forward, 5′-AGCTCCCGTAGGAGAAAATGC-3′, reverse, 5′-CCTGTGGAGACCAAGCGG-3′; FER1L4, forward, 5′-ACCGGAGAGATGTCGAGTGA-3′, reverse, 5′-TCAAAGCGGAACACAAAGCG-3′; GVINP1, forward, 5′-AGAAGCCATGAGTGCAGCTT-3′, reverse, 5′-TTCCAGCAGCCACAGAGATG-3′; NCF1C, forward, 5′-TGTTCCTGGTGAAATGGCAG-3′, reverse, 5′-CTCTGGATTGATCGCCCCTG-3′; NSUN5P2,forward, 5′-CCCCCTTAGATCCGCGCTAT-3′, reverse, 5′-TCGGCATACCCGAGCCA-3′; PLEKHA8P1, forward, 5′-TGGTAAAACATTGCGGCAACA-3′, reverse, 5′-CCCTCTGCATCCCAATACTGAAA-3′; RP9P, forward, 5′-TGAAGGTAAAGACGGAAGCAC-3′, reverse, 5′-CCTCTGTTCCTTGGTCAGTGT-3′; GAPDH, forward, 5′-GAGATGGTGATGGGATTTC-3′, reverse, 5′-GAAGGTGAAGGTCGGAGT-3′; ALU, forward, 5′-GAGGCTGAGGCAGGAGAATCG-3′, reverse, 5′-GTCGCCCAGGCTGGAGTG-3′^45^.

### Functional annotation and pathway analysis for DE mRNAs

The DE mRNAs involved in the ceRNA network were input into WebGestalt (http://www.webgestalt.org/), a functional enrichment analysis web tool to study the Kyoto Encyclopedia of Genes and Genomes (KEGG) pathway^46^ and gene ontology (GO) function of these genes. Then the R package “dplyr” and “ggplot2” were used to analyze the results data and plot the maps of the top 10 most significant items of the KEGG pathway and GO function analysis. The GO functions included biological process (BP), cellular component (CC) and molecular function (MF).

### Ethics approval

Research protocols were approved by the Institutional Review Board of the Peking University Cancer Hospital and Institute. All patients in this study provided written informed consent.

## Supplementary Information

Supplementary Information 1.

Supplementary Information 2.

## Data Availability

The datasets used or analyzed during the current study are available from the corresponding author on reasonable request.

## Abbreviations

CRC: Colorectal cancer
LncRNA: Long noncoding RNA
ceRNA: Competing endogenous RNA
DE genes: Differentially expressed genes
TCGA: The Cancer Genome Atlas
COAD: Colon adenocarcinoma
GEO: Gene Expression Omnibus
qPCR: Quantitative real-time PCR
KEGG: Kyoto Encyclopedia of Genes and Genomes
GO: Gene ontology
BP: Biological process
CC: Cellular component
MF: Molecular function
TF: Transcription factor
CMSs: Consensus molecular subtypes
CRISs: Colorectal cancer intrinsic subtypes
