# Supplementary material for: Pseudogenes and the associated ceRNA network as potential prognostic biomarkers for colorectal cancer
Source: Sci Rep. 2022 Oct 22;12:17787. doi: 10.1038/s41598-022-22768-y (PMC9588006; doi:10.1038/s41598-022-22768-y)
Supplement: Supplementary file 1 — Supplementary Information 1. [file 41598_2022_22768_MOESM1_ESM.pdf]

Supplemental Table 1. The list of DE 31 pseudogenes, 17 miRNAs and 152 mRNAs in the ceRNA network (the name of survival-related genes are in bold).

Gene symbol	logFC	PValue	FDR	Gene symbol	logFC	PValue	FDR
**Up regulated pseudogenes**				**Up regulated mRNAs**			
**PLEKHA8P1**	1.669046	2.36E-28	2.99E-27	OSBPL3	1.7997	1.82E-52	1.11E-50
**RP9P**	1.066084	1.67E-24	1.59E-23	**CCND1**	1.637838	5.22E-50	2.73E-48
C2orf27A	1.87857	4.67E-20	3.23E-19	SCD	2.579664	2.73E-49	1.35E-47
RPLP0P2	1.707331	1.62E-14	7.27E-14	MET	1.783427	2.98E-47	1.32E-45
**NSUN5P1**	1.508603	2.12E-14	9.43E-14	SLC6A6	2.703952	5.49E-47	2.4E-45
MIPEPP3	1.040167	4.6E-13	1.85E-12	PPM1H	2.192963	7.49E-46	3.05E-44
**DDX12P**	1.079841	1.77E-11	6.26E-11	OTX1	5.263449	5.38E-43	1.82E-41
ZNF37BP	1.047852	2.98E-11	1.04E-10	SOX4	1.608656	9.2E-42	2.9E-40
NSUN5P2	1.213042	3.74E-10	1.19E-09	KIAA1549	2.255007	7.85E-41	2.33E-39
**FER1L4**	2.165097	2.65E-09	7.79E-09	DUSP14	1.817861	1.22E-40	3.59E-39
TMEM191A	1.095527	3.45E-08	9.12E-08	SALL4	4.288856	2.04E-37	4.83E-36
OR2I1P	1.573362	2.05E-05	4.17E-05	**RPGRIP1L**	1.353984	2.33E-34	4.56E-33
GAPDHP65	1.093321	0.000119	0.000222	CBX4	1.439166	4.6E-34	8.77E-33
GAPDHP1	1.042733	0.00019	0.000345	VEGFA	1.417921	5.24E-34	9.97E-33
ZDHHC8P1	1.164729	0.000873	0.001465	KIF23	1.412516	1.33E-33	2.48E-32
**Down regulated pseudogenes**				CNN2	1.158564	1.58E-33	2.93E-32
GGTA1P	-2.74297	4.12E-63	4.55E-61	SNTB1	2.081557	2.48E-33	4.51E-32
PIGCP1	-1.53115	5.41E-48	2.45E-46	TGIF2	1.795166	4.12E-33	7.4E-32
MEIS3P1	-2.54972	1.49E-36	3.33E-35	MACC1	2.250083	4.25E-33	7.61E-32
TP73-AS1	-1.81728	3.55E-36	7.71E-35	SCLY	1.327166	1.17E-26	1.31E-25
CMAHP	-1.96278	3.59E-32	5.98E-31	PMAIP1	1.917991	1.44E-25	1.48E-24
NAPSB	-1.87418	8.62E-30	1.2E-28	C2orf15	1.204979	6.7E-24	6.1E-23
ABHD11-AS1	-1.88231	9.92E-27	1.11E-25	MMP11	3.606282	1.45E-23	1.29E-22
**NCF1C**	-2.08156	9.09E-26	9.47E-25	ZDHHC9	1.06263	3E-23	2.61E-22
ANKRD36BP2	-2.63676	4.11E-23	3.54E-22	PLAU	1.83364	5.61E-23	4.79E-22
**GVINP1**	-1.76737	4.59E-23	3.94E-22	SLC7A1	1.045333	1.75E-22	1.45E-21
HMGN2P46	-1.20083	9.28E-16	4.61E-15	**SLC7A6**	1.16013	4.47E-22	3.57E-21
AP000769.1	-1.05134	2.17E-11	7.64E-11	SLC7A11	2.586618	1.67E-21	1.29E-20
RPL41P1	-2.4741	2.49E-09	7.35E-09	**SNAI1**	1.649191	3.02E-21	2.3E-20
AC138123.1	-1.32981	3.27E-08	8.67E-08	FOSL1	3.314619	5.67E-21	4.23E-20
YWHAZP4	-1.31044	8.92E-06	1.88E-05	COL1A1	2.141861	6.77E-20	4.63E-19
YWHAZP5	-1.03007	0.000318	0.000562	SKP2	1.000632	7.5E-19	4.73E-18
**Up regulated miRNAs**				**DNMT3B**	1.433015	5.38E-18	3.17E-17
hsa-miR-140-5p	3.956283	3.14E-39	6.15E-38	ZNF367	1.06801	6.87E-18	4.02E-17
hsa-miR-142-3p	6.644751	6.84E-32	7.02E-31	PLS3	1.161045	2.18E-17	1.23E-16
hsa-miR-429	5.398584	9.54E-32	9.56E-31	EIF4EBP1	1.309117	3.28E-17	1.83E-16
hsa-miR-590-5p	4.91899	9.7E-23	5.61E-22	**OTUB2**	1.611091	3.93E-17	2.17E-16
hsa-miR-455-5p	3.942752	1.13E-20	5.53E-20	MEX3D	1.020138	2.16E-15	1.04E-14
hsa-miR-199b-5p	3.949604	8.66E-19	3.91E-18	DUSP10	1.190889	3.15E-15	1.5E-14
hsa-miR-146b-5p	2.28562	2.71E-12	7.23E-12	AXIN2	1.929588	7.13E-15	3.29E-14
**hsa-miR-217**	4.263241	8.92E-11	2.21E-10	ARID3A	1.70677	1.49E-14	6.73E-14
**hsa-miR-193a-3p**	2.811072	1.22E-10	2.94E-10	METTL26	1.109164	3.07E-13	1.26E-12
hsa-miR-338-3p	2.582417	2.09E-07	4.11E-07	OLR1	2.764851	2.8E-12	1.06E-11
hsa-miR-107	1.153045	3.75E-05	6.06E-05	ONECUT2	2.138664	2.36E-10	7.66E-10
**hsa-miR-34c-5p**	1.476441	0.001967	0.002712	GPCPD1	1.008293	5.29E-10	1.66E-09
**down regulated miRNAs**				KLK10	3.666508	3.01E-09	8.8E-09
hsa-miR-139-5p	-4.90624	2.09E-44	9.41E-43	**FADS1**	1.221632	7.84E-08	2.01E-07
**hsa-miR-125a-5p**	-3.92393	3.84E-40	8.66E-39	**DACH1**	1.524078	9.86E-07	2.29E-06
hsa-miR-129-5p	-5.31729	3.04E-29	2.64E-28	VCAN	1.008896	6.45E-06	1.38E-05
hsa-miR-375	-3.20866	9.14E-11	2.25E-10	MYCN	1.137858	9.12E-05	0.000172
hsa-miR-133b	-2.48455	0.00042	0.000617				

Gene symbol	logFC	PValue	FDR	Gene symbol	logFC	PValue	FDR
**down regulated mRNAs**				**down regulated mRNAs**			
LIFR	-3.75936	6.25E-94	4.03E-91	BMF	-1.25713	2.36E-24	2.22E-23
GFI1	-2.99675	2.08E-89	9.97E-87	MARCKS	-1.27157	3.9E-24	3.62E-23
CNNM2	-1.69158	4.37E-80	1.32E-77	SMAD7	-1.04743	2.31E-23	2.03E-22
NEGR1	-3.10577	1.89E-73	3.55E-71	**SOCS6**	-1.02842	2.95E-23	2.57E-22
**NCAM1**	-3.14862	1.05E-71	1.71E-69	ZEB2	-1.73274	4.4E-23	3.79E-22
**PCSK5**	-2.79471	7.74E-66	1.01E-63	SUN2	-1.11165	5.34E-23	4.56E-22
PDCD4	-2.0661	1.41E-65	1.8E-63	SECISBP2L	-1.05878	1.79E-22	1.48E-21
TEF	-1.8562	3.32E-63	3.7E-61	PDE4D	-1.33879	2.06E-22	1.69E-21
NR5A2	-2.60448	1.33E-62	1.41E-60	RASSF2	-1.47462	3.2E-22	2.59E-21
**HAPLN1**	-2.81401	1.85E-60	1.83E-58	TMEM25	-1.52519	6.22E-22	4.94E-21
CPEB3	-1.44355	2.14E-55	1.57E-53	SHROOM3	-1.03146	1.15E-21	9.03E-21
**GPD1L**	-1.52794	3.14E-51	1.74E-49	DENND5B	-1.26018	3.12E-21	2.38E-20
KAT2B	-1.83737	3.29E-48	1.53E-46	RAB30	-1.12057	3.9E-21	2.94E-20
SLC35G1	-1.42763	1.09E-47	4.91E-46	IL6ST	-1.25213	5.26E-21	3.93E-20
IRF4	-2.94795	9.36E-46	3.78E-44	FGF2	-1.71963	1.83E-20	1.31E-19
RGMA	-2.81351	6.74E-45	2.59E-43	PLEKHO1	-1.3139	1.96E-20	1.4E-19
KLF4	-2.72514	8.33E-45	3.19E-43	**C11orf54**	-1.01027	2.69E-20	1.9E-19
THRB	-2.96107	8.4E-43	2.81E-41	TP53INP1	-1.16789	3.59E-20	2.51E-19
SGK1	-2.40678	4.8E-42	1.56E-40	CAMK2N1	-1.34078	5.44E-19	3.47E-18
**ANKRD33B**	-2.23809	7.92E-42	2.52E-40	HK2	-1.22462	7.58E-19	4.78E-18
**SNCG**	-2.33812	9.86E-41	2.92E-39	PCDH7	-1.61879	8.6E-19	5.39E-18
SEMA6A	-2.86826	1.13E-40	3.32E-39	GNAQ	-1.0252	1.49E-18	9.16E-18
**BCL2L15**	-1.90771	1.19E-40	3.48E-39	GLI3	-1.73725	1.58E-18	9.71E-18
CFL2	-1.93398	1.62E-40	4.75E-39	RPS6KA6	-2.33451	2.45E-18	1.48E-17
**RBM47**	-1.25806	8.22E-40	2.3E-38	**RAB3B**	-1.78797	3.39E-18	2.03E-17
**PPP1R16B**	-2.04992	1.03E-39	2.86E-38	VLDLR	-1.42337	1.58E-17	9.01E-17
KIT	-2.29449	3.67E-39	9.73E-38	ZFPM2	-1.66444	1.77E-17	1E-16
MPP2	-1.96711	2.54E-37	6E-36	DAB2IP	-1.01374	2.16E-17	1.22E-16
ZSWIM6	-1.07424	5.89E-37	1.35E-35	BNC2	-1.68076	2.58E-17	1.45E-16
FOXP2	-2.83541	5.91E-35	1.2E-33	RUNX1T1	-1.53701	5.12E-17	2.8E-16
CPT1A	-1.31993	9.76E-35	1.96E-33	LPP	-1.29441	5.66E-17	3.09E-16
IGSF3	-1.31611	2.11E-34	4.13E-33	PEG10	-2.30817	6.85E-17	3.72E-16
PAG1	-1.79445	6.15E-34	1.16E-32	RET	-1.92298	9.63E-17	5.17E-16
WASL	-1.0876	1.42E-32	2.45E-31	GRAP2	-1.30437	1.62E-16	8.57E-16
PDE3A	-2.55988	3.2E-32	5.36E-31	ANKRD44	-1.00907	1.88E-16	9.95E-16
HCFC2	-1.13598	4.08E-31	6.25E-30	ZEB1	-1.33434	5.06E-16	2.58E-15
RECK	-1.39397	8.79E-31	1.31E-29	AXL	-1.27867	9.28E-16	4.61E-15
FOXN3	-1.32549	4.71E-30	6.63E-29	**MTUS1**	-1.0565	1.03E-15	5.1E-15
RNF125	-1.68926	6.74E-30	9.43E-29	CBX6	-1.31841	2.42E-15	1.16E-14
FAM49A	-1.72629	2.2E-29	2.97E-28	**PDGFRA**	-1.39318	1.31E-14	5.94E-14
FKBP1B	-2.04111	7.69E-29	1.01E-27	**GFI1**	-1.4739	1.36E-14	6.18E-14
TMEM59	-1.07076	1.91E-28	2.44E-27	PCDH19	-2.03705	1.96E-14	8.77E-14
RIMS3	-1.79968	1.05E-27	1.27E-26	FGFR1	-1.23166	1.71E-13	7.13E-13
RPS6KA5	-1.22881	1.47E-27	1.77E-26	**SALL1**	-1.4489	2.22E-13	9.17E-13
TTC28	-1.51055	2.03E-27	2.41E-26	DUSP1	-1.30977	2.66E-12	1.01E-11
CAV1	-1.79746	3.74E-27	4.31E-26	SOX6	-1.24829	2.91E-10	9.34E-10
**ENPP2**	-1.94	5.41E-27	6.17E-26	REEP1	-1.47992	1.35E-08	3.72E-08
EBF1	-1.54622	1.52E-25	1.56E-24	SATB2	-1.23214	1.49E-08	4.1E-08
PLXNA2	-1.21811	4.04E-25	4.01E-24	WASF3	-1.31779	3.11E-08	8.26E-08
DNAJB4	-1.1257	6.19E-25	6.06E-24	GAS1	-1.2225	9.7E-06	2.03E-05
ITPR1	-1.40291	1.63E-24	1.56E-23	**VAV3**	-1.03818	0.000159	0.00029

Supplemental Table 2. Associations between expression level of 7 pseudogenes and clinicopathological factors of patients in TCGA-COAD.

		DDX12P			FER1L4			GVINP1			PLEKHA8P1		
**Characteristic**	**Number**	**Low**	**High**	**Pvalue**	**Low**	**High**	**Pvalue**	**Low**	**High**	**Pvalue**	**Low**	**High**	**Pvalue**
**Age**				0.359			0.108			0.236			0.163
<60	125	67	58		55	70		57	68		56	69	
≥60	328	160	168		172	156		170	158		171	157	
**Gender**				0.965			0.479			0.121			0.886
Male	239	120	119		116	123		128	111		119	120	
Female	214	107	107		111	103		99	115		108	106	
**T stage**				**0.049**			0.789			0.722			**0.023**
T1	12	9	3		5	7		6	6		9	3	
T2	77	45	32		36	41		34	43		35	42	
T3	308	151	157		159	149		158	150		164	144	
T4	56	22	34		27	29		29	27		20	36	
**N stage**				0.745			**0.025**			**0.019**			0.955
N0	266	135	131		145	121		121	145		133	133	
N1+N2	187	92	95		82	105		106	81		94	93	
**M stage**				0.788			**0.018**			**0.006**			0.077
M0	332	175	157		178	154		166	166		175	157	
M1	64	35	29		24	40		44	20		26	38	
**Pathologic stage**				0.473			**0.009**			**0.013**			0.835
Stage I+II	251	132	119		140	111		114	137		128	123	
Stage III+IV	192	94	98		83	109		110	82		96	96	

		NCF1C			NSUN5P2			RP9P		
**Characteristic**	**Number**	**Low**	**High**	**P-value**	**Low**	**High**	**P-value**	**Low**	**High**	**P-value**
**Age**				0.619			0.330			0.848
<60	125	65	60		58	67		62	63	
≥60	328	162	166		169	159		166	162	
**Gender**				0.886			0.965			0.370
Male	239	119	120		120	119		115	124	
Female	214	108	106		107	107		112	102	
**T stage**				0.862			0.868			**0.004**
T1	12	5	7		5	7		5	7	
T2	77	37	40		41	36		42	35	
T3	308	155	153		153	155		166	142	
T4	56	30	26		29	27		16	40	
**N stage**				0.893			0.276			**0.003**
N0	266	134	132		139	127		149	117	
N1+N2	187	93	94		88	99		78	109	
**M stage**				0.083			0.103			**0.046**
M0	332	163	169		177	155		175	157	
M1	64	39	25		27	37		25	39	
**Pathologic stage**				0.652			0.149			**0.005**
Stage I+II	251	124	127		135	116		141	110	
Stage III+IV	192	99	93		90	102		82	110	

Supplemental Table 3. Clinical characteristics of the training and validation cohorts

Characteristics	Total<br>n=453	Training cohort<br>n=227	Validation cohort<br>n=226	P-value
**Age**				0.236
<60	125	57	68	
≥60	328	170	158	
**Gender**				0.278
Male	239	114	125	
Female	214	113	101	
**T stage**				0.393
T1	12	8	4	
T2	77	34	43	
T3	308	159	149	
T4	56	26	30	
**N stage**				0.230
N0	266	127	139	
N1+N2	187	100	87	
**M stage**				1.000
M0	332	166	166	
M1	64	32	32	
**Pathologic stage**				0.330
Stage I+II	251	119	132	
Stage III+IV	192	100	92	

Supplemental Table 4. The 5-pseudogene risk score model

Pseudogene	Coef	Exp(coef)	Se(coef)	z	P-value
DDX12P	0.002045	1.002047	0.001149	1.780	0.0751
NCF1C	0.003879	1.003887	0.001597	2.429	0.0151
PLEKHA8P1	0.003856	1.003863	0.001908	2.020	0.0433
RP9P	0.001913	1.001915	0.001161	1.647	0.0995
YWHAZP4	-0.006358	0.993662	0.003542	-1.795	0.0726
