# Supplementary material for: Pseudogenes and the associated ceRNA network as potential prognostic biomarkers for colorectal cancer
Source: Sci Rep. 2022 Oct 22;12:17787. doi: 10.1038/s41598-022-22768-y (PMC9588006; doi:10.1038/s41598-022-22768-y)
Supplement: Supplementary file 2 — Supplementary Information 2. [file 41598_2022_22768_MOESM2_ESM.pdf]

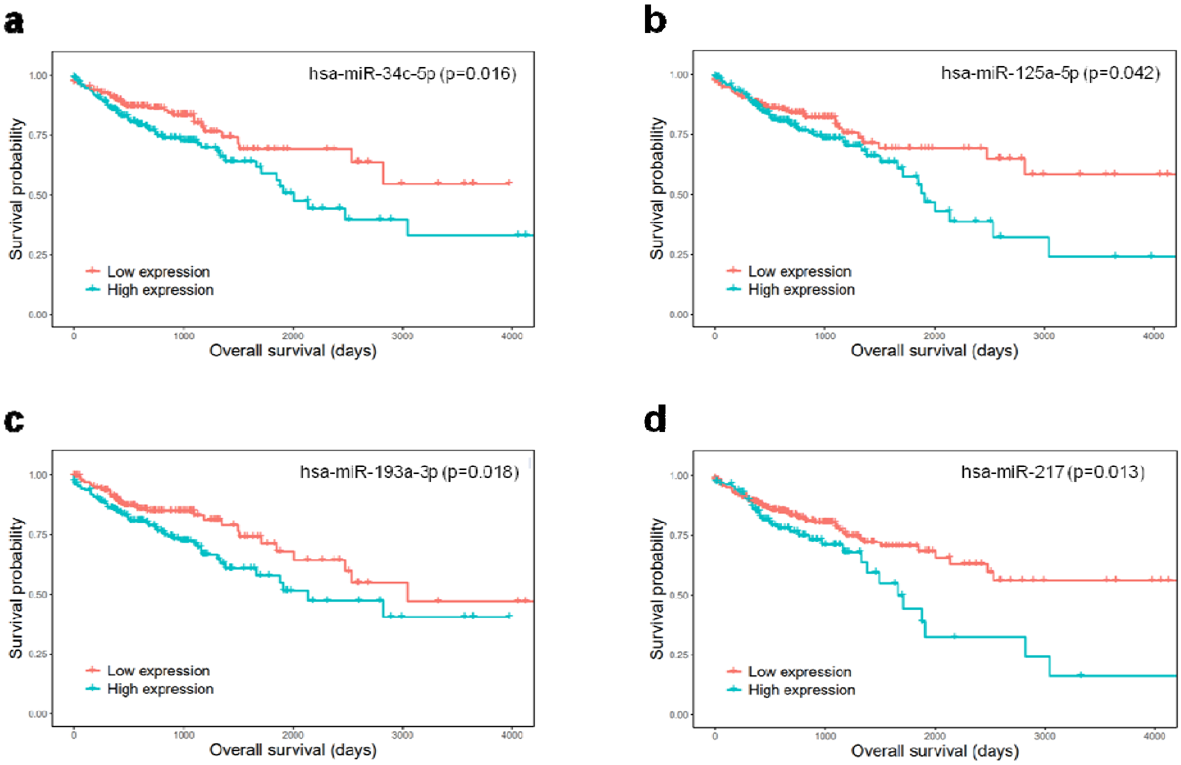

Supplemental Figure 1. Kaplan-Meier curve analysis of survival-related DE miRNAs in the ceRNA network for CRC patients from TCGA-COAD.

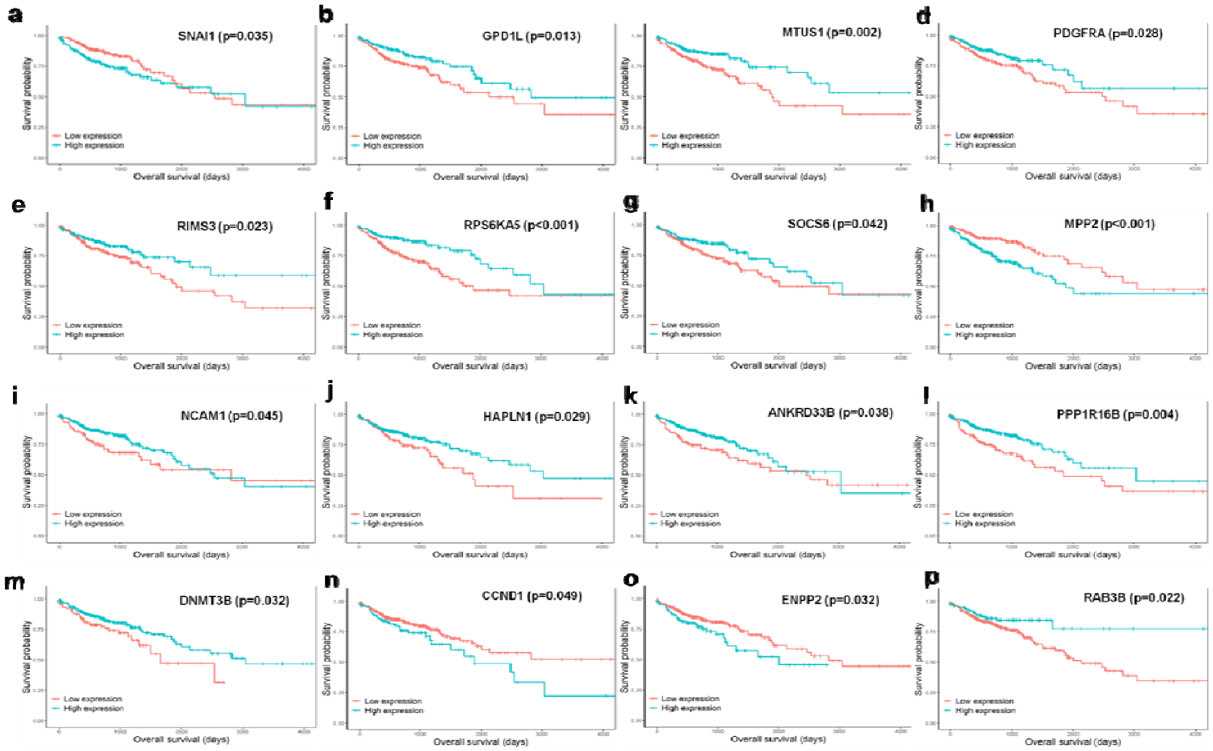

Supplemental Figure 2. Kaplan-Meier curve analysis of some survival-related DE mRNAs in the ceRNA network for CRC patients from TCGA-COAD.

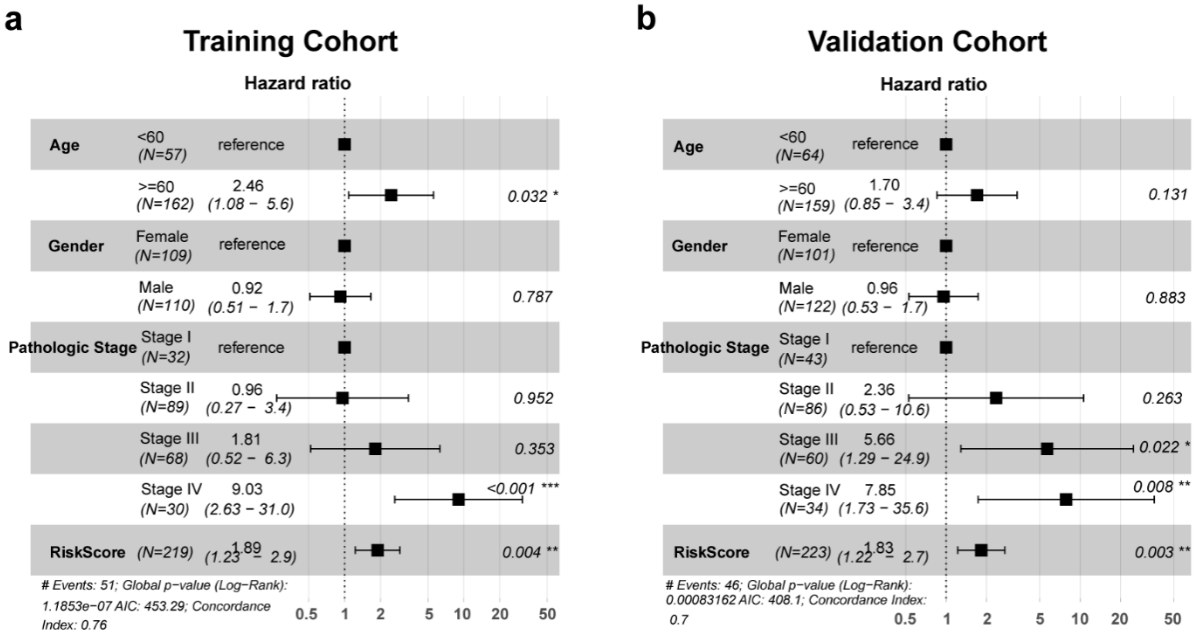

Supplemental Figure 3. Multivariate prognostic analysis of the risk score of 5-pseudogene prognostic signature integrated with clinical characteristics.

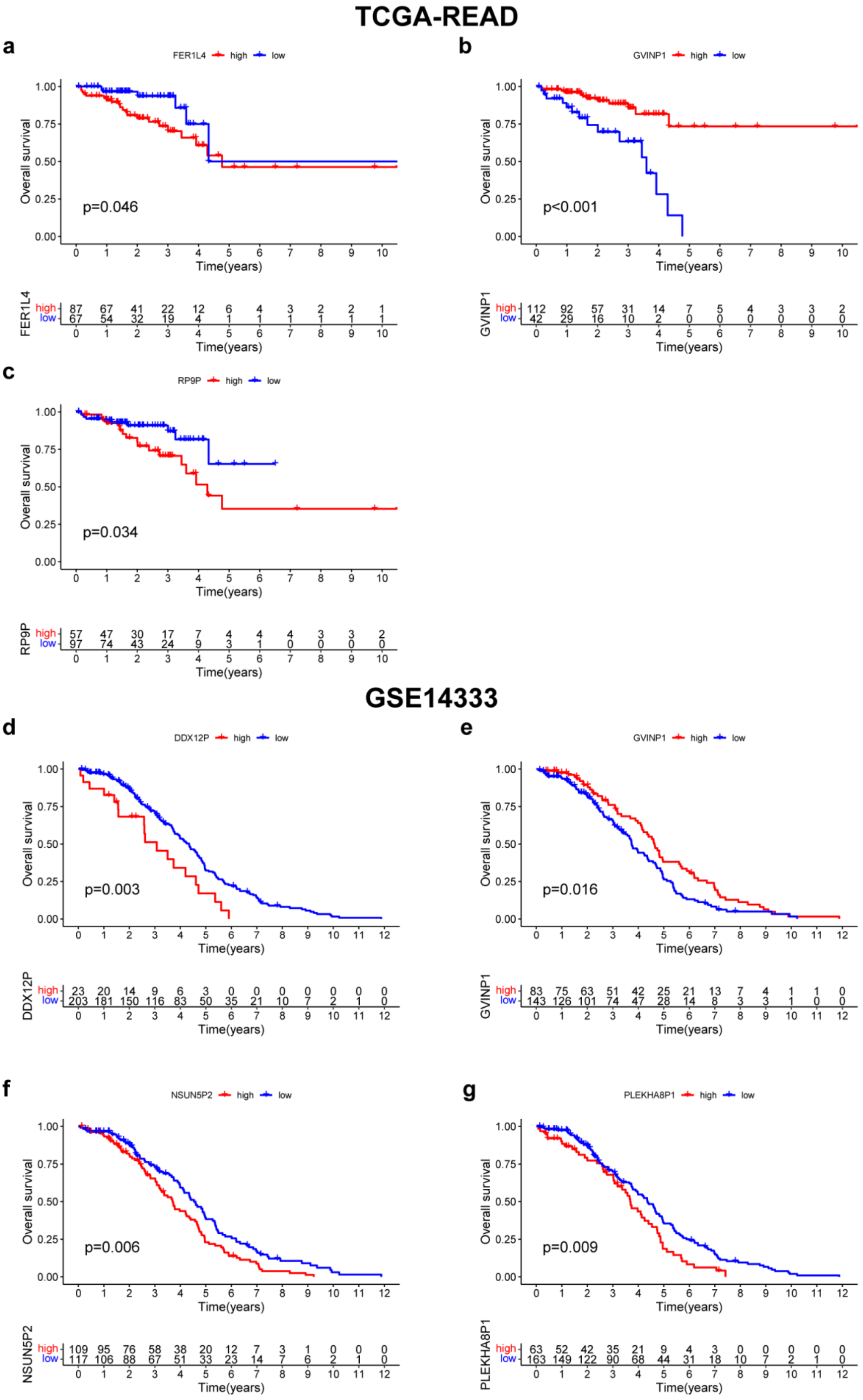

Supplemental Figure 4. Kaplan-Meier curve analysis of pseudogenes for samples from TCGA-READ and GSE14333 datasets.

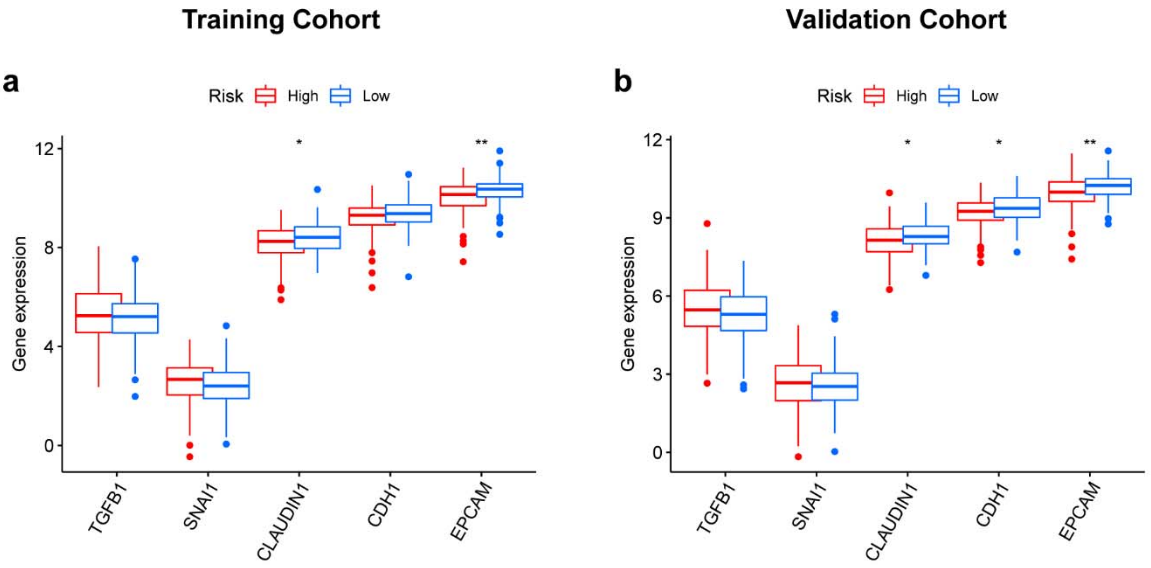

Supplemental Figure 5. The differential expression of stromal and epithelial genes in the high- and low-risk groups in the training and validation cohorts.
